# RNF43 is a novel tumor-suppressor and prognostic indicator in clear cell renal cell carcinoma

**DOI:** 10.32604/or.2022.03458

**Published:** 2022-08-01

**Authors:** DAWEI ZHU, LEI ZHANG, XIAOKAI SHI, SHENGLIN GAO, CHUANG YUE, LIFENG ZHANG, YU BAI, QIFENG WANG, ATSUSHI OKADA, TAKAHIRO YASUI, CHAO WANG, XINGANG CUI, LI ZUO

**Affiliations:** 1 Department of Urology, The Affiliated Changzhou No. 2 People’s Hospital of Nanjing Medical University, Changzhou, China; 2 Department of Pathology, Fudan University Shanghai Cancer Center, Shanghai, China; 3 Department of Nephro-Urology, Nagoya City University Graduate School of Medical Sciences, Aichi, Japan; 4 Department of Urinary Surgery, Gongli Hospital, Second Military Medical University (Naval Medical University), Shanghai, China; 5 Department of Urinary Surgery, The Third Affiliated Hospital of Naval Medical University (Eastern Hepatobiliary Surgery Hospital), Shanghai, China

**Keywords:** Clear cell renal cell carcinoma, RNF43, Prognosis, YAP, Tumor progression

## Abstract

Identifying prognostic indicators of clear cell renal cell carcinoma (ccRCC) and elucidating the mechanisms underlying ccRCC progression are crucial for improving ccRCC patient prognosis. This study investigated the clinical significance and biological role of Ring finger protein 43 (RNF43) in ccRCC. Two independent cohorts of patients with ccRCC were employed to determine the prognostic significance of RNF43 by immunohistochemistry and statistical analyses. *In vitro* and *in vivo* experiments, RNA-seq, and other techniques were used to determine the biological role of RNF43 in ccRCC and related molecular mechanisms. RNF43 expression was commonly decreased in ccRCC specimens, and low expression of RNF43 indicated a higher TNM stage, SSIGN score, and WHO/ISUP grade and short survival in patients with ccRCC. Additionally, RNF43 overexpression suppressed the proliferation, migration, and targeted drug resistance of ccRCC cells, while the knockdown of RNF43 enhanced these characteristics of ccRCC. RNF43 knockdown activated YAP signaling by decreasing YAP phosphorylation by p-LATS1/2 and increasing the transcription and nuclear distribution of YAP. By contrast, RNF43 overexpression showed the opposite effects. Decreasing YAP abolished the effect of RNF43 knockdown in promoting the malignant features of ccRCC. Additionally, restoring RNF43 expression suppressed the resistance of the targeted drug pazopanib in *in vivo* orthotopic ccRCC. Furthermore, combining the expression of RNF43 and YAP with TNM stage or the SSIGN score exhibited greater accuracy than any of these indicators alone in assessing the postoperative prognosis of ccRCC patients. In summary, our study identified a novel tumor suppressor, RNF43, which is also a prognostic indicator and potential target for ccRCC.

## Introduction

Clear cell renal cell carcinoma (ccRCC) is the most common type of kidney cancer [1]. ccRCC patients generally receive surgical therapy, while those with advanced ccRCC are treated with targeted drugs because of the insensitivity of ccRCC to chemotherapy and radiotherapy [2]. However, ccRCC usually progresses even after various therapies, leading to a poor prognosis in ccRCC patients [3]. Reliable prognostic indicators of ccRCC have not been identified, and the molecular mechanisms underlying ccRCC progression have not been elucidated.

Ring finger protein 43 (RNF43), an E3 ubiquitin ligase, is involved not only in the ubiquitination and stabilization of p53 [4] but also in the cell cycle [5]. Furthermore, RNF43 exerts suppressive effects in various cancers, such as colorectal cancer, gastric cancer, and endometrial cancer [6,7]. RNF43 mutations frequently appear in these cancers, and the downregulation of RNF43 predicts a poor prognosis in patients [8]. RNF43 inhibits the Wnt pathway by ubiquitinating Wnt receptors of the Frizzled (Fzd) family or sequestering the transcription factor TCF4 to the nuclear membrane, thereby impairing Wnt-target gene expression [9,10]. Additionally, RNF43 inhibits gastric cancer stem-like cells by inactivating Wnt/β-catenin signaling [11]. However, the expression and biological effects of RNF43 in ccRCC remain unknown.

The Hippo pathway is indicated to be involved in homeostasis, with roles such as modulating cell proliferation and differentiation in developing organs [12]. Inactivation of the Hippo pathway results in unlimited cell growth and tumors [13]. The core of the Hippo pathway comprises a kinase cascade and transcription coactivators, including Yes-associated protein (YAP), a core transcription coactivator that is negatively regulated by upstream kinases such as large tumor suppressor 1 and 2 (LATS1/2). YAP exerts a protumoral function in various cancers, including ccRCC, by translocating into the nucleus to bind to members of the TEA domain (TEAD) transcription factor family, thereby inducing various genes [14]. However, the related mechanisms remain unclear.

In our present study, 320 ccRCC patients from two independent cohorts were employed to examine the clinical significance of RNF43. Additionally the biological functions and related mechanisms of RNF43 were determined in ccRCC cells and an *in vivo* pazopanib-resistant orthotopic ccRCC model. Our results demonstrate that RNF43 is a novel tumor suppressor in ccRCC that inhibits YAP transcription and nuclear localization. Furthermore, greater accuracy is achieved by integrating the expression of RNF43 and YAP with the current clinical indicators to predict the survival of ccRCC patients.

## Materials and Methods

### Analysis of public databases

Datasets from The Cancer Genome Atlas (TCGA) were downloaded from National Cancer Institute GDC Data Portal (https://portal.gdc.cancer.gov), and 533 samples from ccRCC patients were filtered out. Wilcox test was used to detect the RNF43 expression in ccRCC.

### Patients and specimens

Three hundred patients who were pathologically diagnosed with ccRCC between 2010 and 2014 from cohort 1 (the Affiliated Changzhou No. 2 People’s Hospital; n = 193) and cohort 2 (Fudan University Shanghai Cancer Center; n = 127) were used in the present study. The study followed the reporting recommendations for studies on prognostic tumor markers (REMARK) [15]. The study was also approved by the institutional ethical review boards of the hospitals, and written informed consent was obtained from all ccRCC patients. The clinicopathological features of all the ccRCC patients are listed in Suppl. Table S1. Another 48 paired ccRCC specimens were used for real-time PCR.

### Real-time PCR assays

Real-time PCR, which was performed as reported previously [16], were performed using the SYBR Green Real-Time PCR Master Mix (QPK201; Toyobo, Osaka, Kansai, Japan) and an ABI PRISM 7300HT Sequence Detection System. The primer sequences used in the study were as follows: *RNF43* (forward primer, 5`-AGCATGAGTGGTGGCCACCAG-3’; reverse primer, 5’-ATCTCACACAGCCTGTTCAC-3’) and *GAPDH* (forward primer, 5’-GGAAGGTGAAGGTCGGAGT-3’; reverse primer, 5’-CCTGGAAGATGGTGATGGG-3’). The results were first normalized to GAPDH expression. Second, the fold change relative to the mean value was determined by 2^−ΔΔCt^.

### Immunohistochemistry (IHC)

IHC experiments were performed as described in our study [17]. IHC assays were performed using an UltraSensitive Streptavidin Peroxidase Kit (KIT-9710; Maixin Biotechnologies, Beijing, China) according to the manufacturer’s instructions. The following primary antibodies were used: rabbit anti-RNF43 antibody (ab217787; Abcam, Cambridge, MA, USA) and rabbit anti-YAP antibody (ab52771). RNF43 staining was scored using an H-score of 0–3 intensity multiplied by the percentage of positive cells (range 0–300), which was described in our study [17].

### Western blot analysis

Western blot assays were performed as described in our previous study [17]. Briefly, cultured cells or ccRCC tissues were lysed in RIPA lysis buffer, and protein concentrations were determined using the BCA assay kit according to the manufacturer’s protocols (Thermo Fisher Scientific, Waltham, MA, USA). The nuclear and cytoplasmic proteins from ccRCC cells were extracted using an NE-PER Nuclear and Cytoplasmic Extraction Kit (#78899; Thermo Scientific). The primary antibodies used were rabbit anti-RNF43 antibody (ab84125; Abcam), rabbit anti-LATS1 + LATS2 antibody (ab70565), rabbit anti-LATS1 + LATS2 (phospho T1079 + T1041) antibody (ab111344), rabbit anti-YAP antibody (ab52771), rabbit anti-YAP (phospho S127) antibody (ab76252), rabbit anti-GAPDH antibody (#5174), rabbit anti-β-actin antibody (#58169), and rabbit anti-histone H3 antibody (#4499), all of which were from Cell Signaling Technology (Danvers, MA, USA).

### Cell culture

The cell lines were obtained from the Cell Bank of the Type Culture Collection of the Chinese Academy of Sciences (Shanghai, China). 786-O, 769-P, and OS-RC-2 cells were cultured in RPMI-1640 medium (22400-089; Gibco/Thermo Fisher). ACHN cells were cultured in Minimum Essential Medium (11095-080; Gibco). HK-2 cells were cultured in high-glucose Dulbecco’s modified Eagle’s medium (DMEM) (11995-065; Gibco). The above culture media were supplemented with 1% penicillin/streptomycin (Gibco) and fetal bovine serum (FBS; 10%; Gibco). The pazopanib-resistant or sunitinib-resistant 786-O cells were cultured in RPMI-1640 medium with 10% (v/v) FBS and the targeted drug pazopanib (8 µM) or sunitinib (10 µM), respectively.

All the cell lines were authenticated by short tandem repeat (STR) profiling, examined for mycoplasma contamination using a Mycoplasma Detection Kit (Selleck Chemicals, Houston, TX, USA), and cultured within 40 passages.

### Gene knockdown and overexpression

Gene knockdown and overexpression were performed in our study [17]. 786-O and 769-P cells were transduced with RNF43-overexpressing lentiviral vectors, shRNA-expressing lentivirus (sh-RNF43) or their respective control lentivirus. The sequences for lentiviral vectors with RNF43-overexpressing and shRNAs (sh-RNF43) are listed in Suppl. Table S2. siRNA silencing was performed using Lipofectamine 3000 reagents (L3000015, Invitrogen, Carlsbad, CA, USA), and the sequence of si-YAP is shown in Suppl. Table S2.

### Cell proliferation and apoptosis

Proliferation assays were performed using a CCK-8 kit (CK-04; Dojindo, Kumamoto, Kyushu, Japan), which was used in our study [17]. The proliferation rates are shown as proportions of the control value. Apoptotic assays were performed using annexin-V and propidium iodide (PI) staining (A13201; Invitrogen), which was examined by flow cytometry using a Cyan ADP Sorter (Beckman, Brea, CA, USA).

### Migration assays

Migration experiments were performed in Transwell chambers (Millipore, Billerica, MA, USA), which were used in our study [17]. Briefly, 1 × 10^4^ 786-O or 769-P cells were seeded into the upper chamber of each uncoated Transwell with RPMI-1640 medium only. RPMI-1640 medium with 20% FBS was placed in the lower chamber. After 36 h or 48 h, the noninvasive ccRCC cells in the upper chamber were removed with a cotton swab, while the ccRCC cells on the lower surface of the membrane were fixed with 4% paraformaldehyde fix solution (E672002; Sangon Biotech, Shanghai, China) and stained with crystal violet (E607309; Sangon Biotech).

### RNA-Seq and analysis

Base calling was performed using the Illumina BaseSpace platform and the FASTQ Generation Application. Read sequences were aligned to the human reference genome GRCh37 using tophat2 (v2.1.1) with GENCODE version 24 gene models. The gene expression levels were inferred from BAM files using htseq-count from HTSeq (v1.3.0). Differential expression analysis was performed using DESeq2 (v1.12.4), and the enrichment of Gene Ontology and Pathway were examined using the R package goProfiles (v3.6).

### Animal experiments

For the subcutaneous tumor formation assay, 786-O cells with RN43 knockdown without or with YAP silencing (5 × 10^6^ cells in 100 μl of PBS) were injected subcutaneously into nude mice, which were euthanized 6 weeks after inoculation. For subcapsular renal tumor formation assays, 6-week-old male NOD-SCID mice were anesthetized, and then a 50-μl Matrigel/medium (1:1) suspension containing 1 × 10^7^ 786-O-PR-luc cells from different groups was inoculated under the renal capsule. The mice were sorted into three groups three weeks post-implantation: the mice injected with 786-O-PR cells were treated with pazopanib (80 mg/kg) or normal saline, and those injected with RNF43-overexpressing 786-O-PR cells were treated with pazopanib (80 mg/kg). The mice were sacrificed eight weeks post-injection. The specimens were removed and fixed in 10% buffered formalin solution. The experimental animal procedures followed the Animal Care and Use Committee of the Affiliated Changzhou No. 2 People’s Hospital of Nanjing Medical University.

### Statistical analysis

The results were expressed as the means±standard deviation (SD) of three independent experiments. Statistical differences between variables were analyzed by the two-tailed Wilcoxon test, and *p* < 0.05 was considered significant. Time-dependent receiver operating characteristic (ROC) analysis computed using the “time ROC” package was used to determine the best cut-off values of RNF43 and YAP AUC. Survival curves were depicted using the Kaplan-Meier method and analyzed by the log-rank test. Variables with *p* < 0.1 in univariate analysis were included in multivariate Cox proportional hazards analysis. The prognostic accuracy was assessed by Harrell’s concordance index (c-index). All the statistical analyses and graphs were performed using GraphPad Prism software (version 8.2.0) and R software (version 3.5.3).

## Results

### RNF43 is downregulated in ccRCC specimens, and low RNF43 expression predicts malignant features of ccRCC

**Figure 1:**
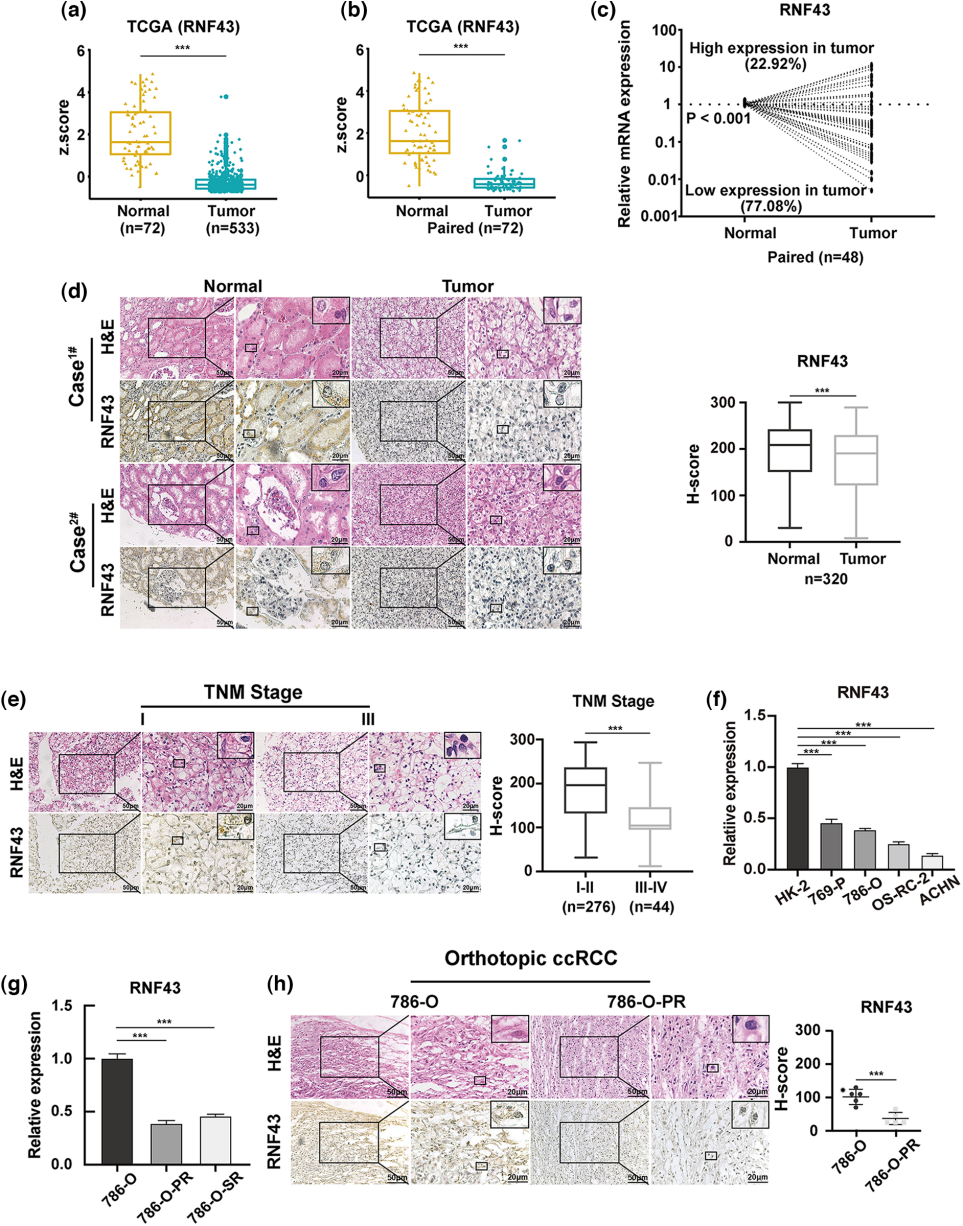
RNF43 is downregulated in ccRCC specimens, and low RNF43 expression predicts malignant features of ccRCC. (a) The expression of RNF43 was analyzed in normal renal tissues (n = 72) and ccRCC tissues (n = 533) from TCGA datasets. (b) The expression of RNF43 was compared between paired tumor and normal tissues (n = 72) in TCGA ccRCC datasets. (c) The expression of RNF43 in matched ccRCC and normal samples was determined by real-time PCR (n = 48). (d) Representative images of H&E staining and IHC staining for RNF43 in ccRCC tissues and adjacent tissues are shown (scale bar = 50 μm, 200× and 20 μm, 400×), and the expression level of RNF43 was calculated using the H-score. (e) Representative images of H&E and IHC staining for RNF43 in ccRCC specimens with different TNM stages are presented (scale bar = 50 μm, 200× and 20 μm, 400×). (f) Real-time PCR was performed to detect the expression of RNF43 in various ccRCC cell lines (786-O, 769-P, OS-RC-2, ACHN) and a normal cell line (HK-2). (g) Real-time PCR was conducted to determine the expression of RNF43 in 786-O-SR or 786-O-PR cells and control naïve 786-O cells. (h) Representative images of H&E and IHC staining for RNF43 in 786-O-derived pazopanib-resistant orthotopic tumors and naïve orthotopic tumors are shown (scale bar = 50 μm, 200× and 20 μm, 400×). ***p < 0.001.

The Cancer Genome Atlas (TCGA) database was first employed to analyze the expression of RNF43 in ccRCC specimens, demonstrated that the mRNA expression of RNF43 was lower in ccRCC samples than in adjacent tissues (Figs. 1a and 1b). Real-time PCR also confirmed that lower RNF43 mRNA expression was observed in most ccRCC specimens than in adjacent tissues (37/48) (Fig. 1c). Additionally, an immunohistochemistry (IHC) experiment was employed in ccRCC specimens (*n* = 320), demonstrating that most ccRCC samples showed decreased RNF43 expression compared with paired normal tissues (254/320) (Fig. 1d). These results indicate that RNF43 expression is decreased in ccRCC.

Next, to examine whether RNF43 was associated with disease progression in ccRCC, IHC assays were used to demonstrate that lower RNF43 expression was found in ccRCC specimens with a higher TNM stage than in those with a lower TNM stage (Fig. 1e). Furthermore, real-time PCR showed that the expression of RNF43 was upregulated in HK-2 (a normal renal cell line) and local ccRCC cell lines and decreased in ACHN (a metastatic cell line) (Fig. 1f). Additionally, lower RNF43 expression was observed in pazopanib- or sunitinib-resistant ccRCC cell lines (786-O-PR or 786-O-SR) and pazopanib-resistant orthotopic tumors (which were described in our previous studies [17,18]) than in their corresponding naïve controls (Figs. 1g and 1h). The results demonstrated that RNF43 expression is negatively associated with the aggressive characteristics of ccRCC.

### Low expression of RNF43 is associated with an unfavorable prognosis in ccRCC patients

**Figure 2:**
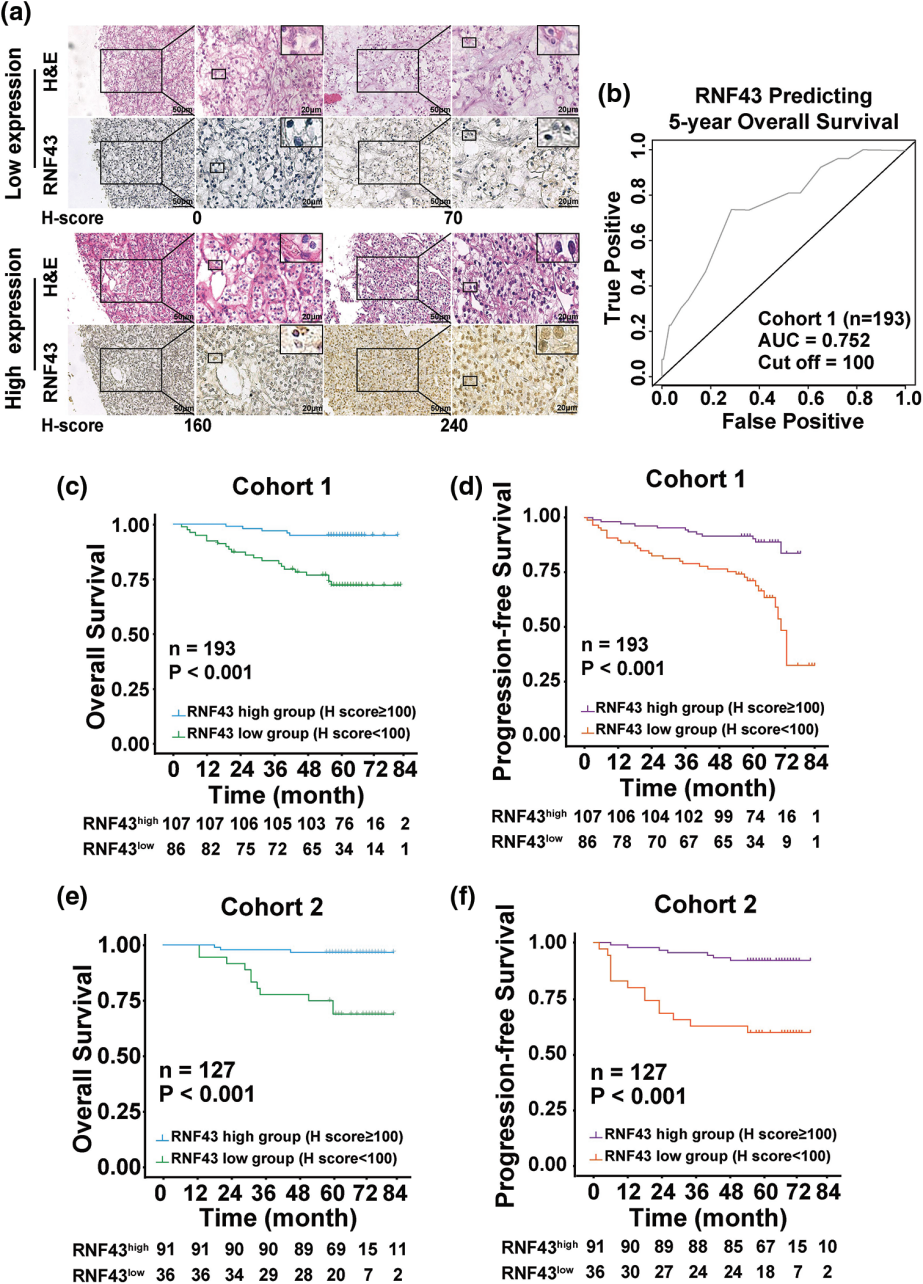
Low expression of RNF43 is associated with an unfavorable prognosis in ccRCC patients. (a) Representative H&E and IHC staining for RNF43 in ccRCC tissues from the RNF43^low^ and RNF43^high^ groups (scale bar = 50 μm, 200× and 20 μm, 400×). (b) Time-dependent ROC analysis was conducted to evaluate the optimum cut-off value of RNF43 in cohort 1. (c–f) Kaplan-Meier curves for the OS and PFS of ccRCC patients were compared between the RNF43^low^ and RNF43^high^ groups in cohort 1 (n = 193; c, d) and cohort 2 (n = 127; e, f).

We next determined whether RNF43 expression predicted the ccRCC patients’ postoperative prognosis. IHC assays were performed in two cohorts of ccRCC patients (Fig. 2a), and then the optimal cut-off value was calculated by time-dependent receiver operating characteristic (ROC) analysis to divide ccRCC patients in cohort 1 (Fig. 2b). According to the optimal cut-off value, ccRCC patients in cohort 1 were divided into the RNF43^low^ and RNF43^high^ groups. Relative to the RNF43^high^ patients, the RNF43^low^ patients presented advanced TNM stages, higher WHO/ISUP grades, and higher stage, sign, grade, and necrosis (SSIGN) scores (Table 1). Additionally, Kaplan-Meier survival analysis was performed to show that the RNF43^low^ group exhibited a shorter overall survival (OS) and progression-free survival (PFS) than the RNF43^high^ group (Figs. 2c and 2d). Additionally, using the cut-off value of RNF43 derived from cohort 1, assessment of cohort 2 revealed that the RNF43^low^ group showed unfavorable clinicopathological characteristics and a shorter OS and PFS than the RNF43^high^ group (Figs. 2e and 2f; Table 2).

**Table 1:** The correlation between RNF43 expression and clinicopathologic characteristics of patients with clear cell renal cell carcinoma in cohort 1

Characteristic	RNF43		Sum (193)	P* value
	Low expression (n = 86)	High expression (n = 107)		
**Age**				0.194
<60	39	59	98	
≥60	47	48	95	
**Gender**				0.124
Male	52	77	129	
Female	34	30	64	
**WHO/ISUP Grading**				0.070
I–II	49	75	124	
III–IV	37	32	69	
**TNM stage**				<0.001*
I–II	59	99	158	
III–IV	27	8	35	
**SSIGN**				<0.001*
0–4	65	101	166	
≥5	21	6	27	
**YAP**				<0.001*
Low expression	37	77	114	
High expression	49	30	79	

Note: *: Statistical significance was calculated by chi-square test or fisher’s exact test for categorical/binary measures.

**Table 2:** The correlation between RNF43 expression and clinicopathologic characteristics of patients with clear cell renal cell carcinoma in cohort 2

Characteristic	RNF43		Sum (127)	P* value
	Low expression (n = 36)	High expression (n = 91)		
**Age**				0.457
<60	14	42	56	
≥60	22	49	71	
**Gender**				0.247
Male	25	72	97	
Female	11	19	30	
**WHO/ISUP Grading**				0.042*
I–II	18	63	81	
III–IV	18	28	46	
**TNM stage**				<0.001*
I–II	28	90	118	
III–IV	8	1	9	
**SSIGN**				<0.001*
0–4	28	90	118	
≥5	8	1	9	
**YAP**				0.003*
Low expression	18	70	88	
High expression	18	21	39	

Note: *: Statistical significance was calculated by chi-square test or fisher’s exact test for categorical/binary measures.

Furthermore, the two independent cohorts of ccRCC patients were combined and then randomly divided into a training cohort (n = 160) and validation cohort (n = 160) at a 1:1 ratio to validate the above results. As shown in Suppl. Tables S3 and S4, a low expression level of RNF43 predicted advanced TNM stage, a higher WHO/ISUP grade and SSIGN score, and a worse OS and PFS in ccRCC patients in the training and validation cohorts (Suppl. Figs. S2A–S2D). These findings demonstrated that RNF43 expression represents a potential prognostic indicator for ccRCC patients.

### RNF43 inhibits the proliferation, migration, and pazopanib resistance of ccRCC

**Figure 3:**
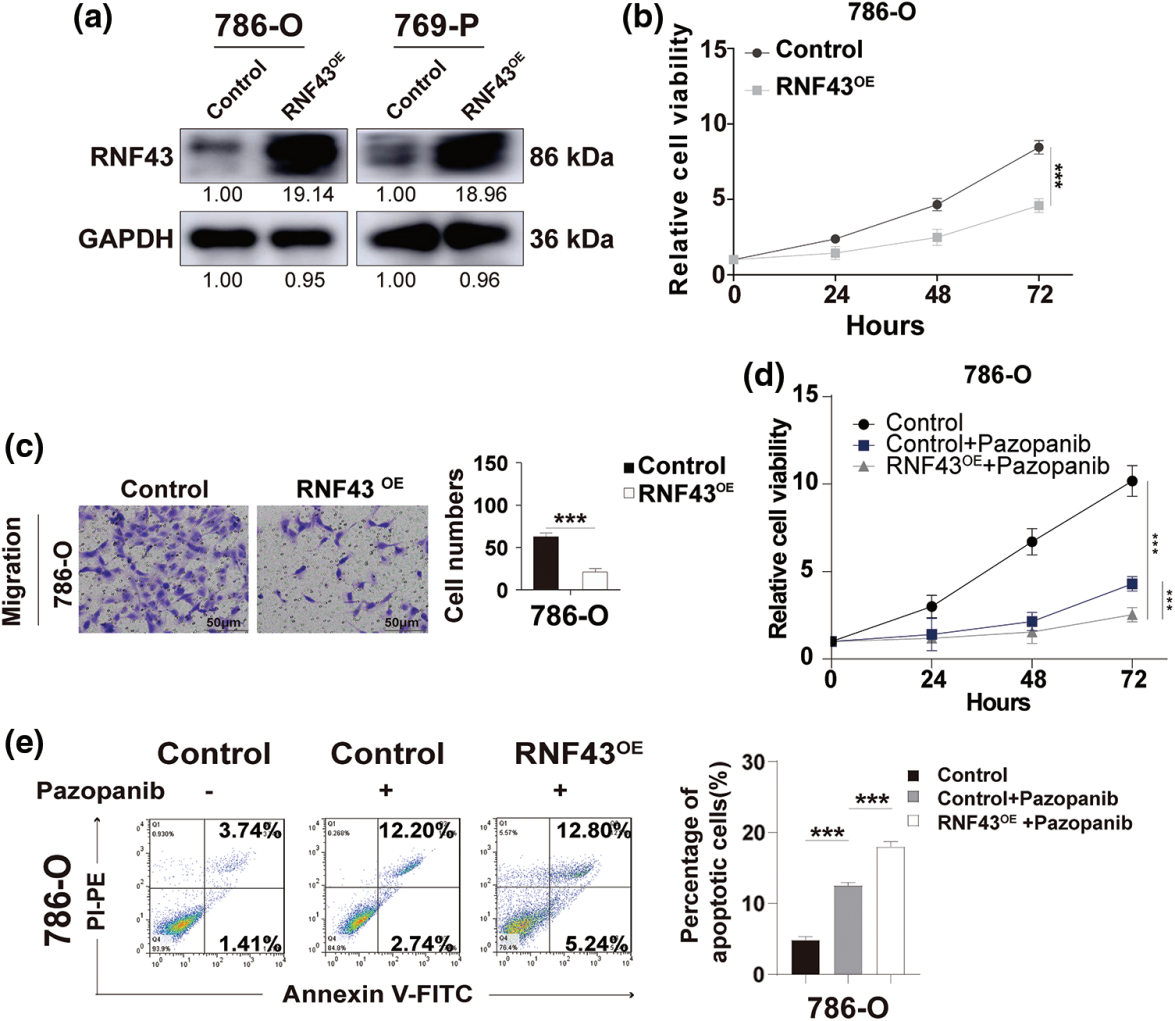
Overexpression of RNF43 inhibits the proliferation, migration, and pazopanib resistance of ccRCC. (a) Western blot assays were performed to detect the expression of RNF43 in 786-O or 769-P cells with or without RNF43 overexpression (RNF43^OE^). (b) CCK-8 proliferation assays were employed to determine the proliferation of 786-O cells without or with RNF43 overexpression(RNF43^OE^). (c) Representative images and statistical analysis of migration assays in 786-O cells without or with RNF43 overexpression(RNF43^OE^) are presented (scale bar = 200 µm). (d, e) 786-O cells without and with RNF43 overexpression were treated with pazopanib (3 μM) for 36 hours, cell proliferation was examined using CCK-8 assays (d), and cell apoptosis was detected using flow cytometry assays (e). **p < 0.01 (n = 3); NS, no significance.

Given the inverse relationship between RNF43 expression and disease progression and the prognosis of ccRCC patients, we next examined whether RNF43 exerts suppressive effects in ccRCC. Lentivirus-derived RNF43 vectors were stably generated in 786-O and 769-P cells (Fig. 3a). First, CCK-8 proliferation experiments were employed to show that RNF43-overexpressing 786-O or 769-P cells exhibited decreased cell proliferation in contrast to that of control ccRCC cells (Fig. 3b; Suppl. Fig. S3a). Next, Transwell assays were employed to reveal fewer migrated ccRCC cells in RNF43-overexpressing 786-O and 769-P cells than in control cells (Fig. 3c; Suppl. Fig. S3b). Given the finding of an inverse relationship between RNF43 expression and pazopanib resistance in ccRCC, we examined whether RNF43 regulated pazopanib resistance in ccRCC. Under pazopanib treatment, RNF43-overexpressing 786-O or 769-P cells showed inhibited proliferation and increased apoptosis compared with the control cells, suggesting that ccRCC cells with RNF43 overexpression were sensitive to the targeted drug (Figs. 3d and 3e; Suppl. Figs. S3c and S3d). These data indicated that RNF43 suppresses the malignant features of ccRCC cells.

**Figure 4:**
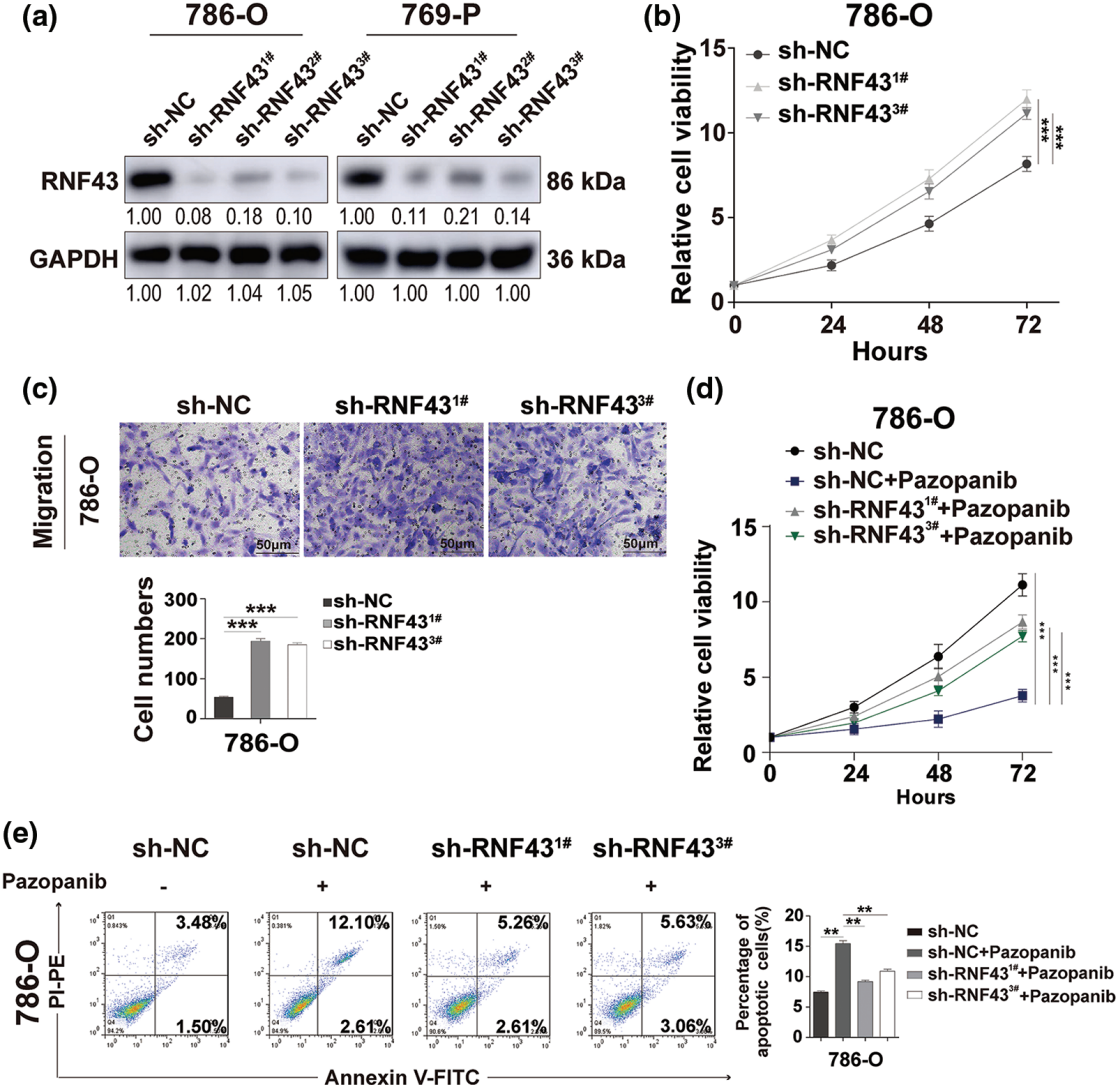
Knock down the expression of RNF43 facilitates the proliferation, migration, and pazopanib resistance of ccRCC. (a) Western blot assays were performed to examine the expression of RNF43 in 786-O or 769-P cells with or without RNF43 knockdown. (b) CCK-8 proliferation assays were used to detect the proliferation of 786-O cells without or with RNF43 knockdown. (c) Representative images and statistical analysis of the migration assays in 786-O cells without or with RNF43 knockdown are shown (scale bar = 200 µm). (d, e) 786-O cells without and with RNF43 knockdown were treated with pazopanib (3μM) for 36 h, cell proliferation was examined by CCK-8 assays (d), and cell apoptosis was detected by flow cytometry assays (e). **p < 0.01 and ***p < 0.001 (n = 3).

To validate the biological role of RNF43 in ccRCC, RNF43 was knocked down in 786-O and 769-P cells by lentivirus-delivered short hairpin RNAs (shRNAs) (Fig. 4a). The CCK-8 proliferation assay revealed a significant increase in the cell proliferation rate in 786-O or 769-P cells with RNF43 knockdown in contrast to that in control cells (Fig. 4b; Suppl. Fig. S3e). Additionally, using Transwell assays, RNF43 knockdown-786-O or 769-P cells presented enhanced migration abilities compared with those of control ccRCC cell lines (Fig. 4c; Suppl. Fig. S3f). Additionally, pazopanib resulted in enhanced proliferation and fewer apoptotic events in 786-O or 769-P cells with RNF43 knockdown than in control ccRCC cells (Figs. 4d and 4e; Suppl. Figs. S3g and S3h). The results indicated that the knockdown of RNF43 facilitates the proliferation, migration, and pazopanib resistance of ccRCC.

### RNF43 inhibits the malignant features of ccRCC by suppressing YAP signaling

**Figure 5:**
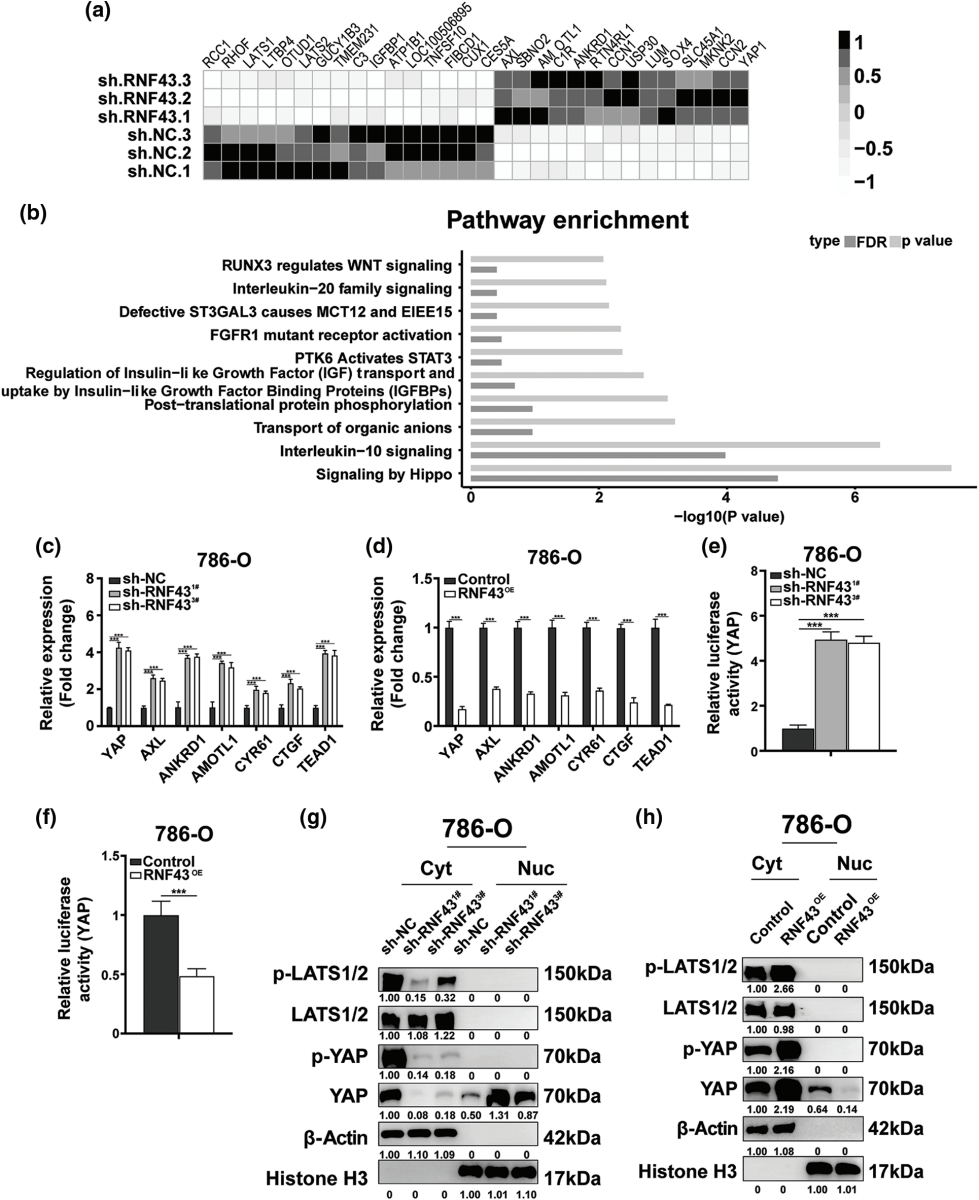
RNF43 inhibits the malignant features of ccRCC by suppressing YAP signaling. (a, b) RNA sequencing was performed in 786-O cells with or without RNF43 knockdown. A heatmap depicting the significantly differentially expressed genes (a) and an analysis of differentially expressed pathways (b) are presented. (c, d) Real-time PCR was performed to examine the expression of YAP, AMOTL1, CTGF, AXL, CYR61, ANKRD1, and TEAD1 in 786-O cells without or with RNF43 knockdown (c) or with RNF43 overexpression (RNF43^OE^) (d). (e, f) Luciferase assays were performed to determine the transcriptional activity of YAP in 786-O cells without or with RNF43 knockdown (e) or with RNF43 overexpression (RNF43^OE^) (f). (g, h) Western blot analysis of p-LATS1/2, LATS1/2, p-YAP, and YAP in cytoplasmic (Cyt) and nuclear (Nuc) fractions of 786-O cells without or with RNF43 knockdown (g) or with RNF43 overexpression (RNF43^OE^) (h). β-Actin and histone H3 were used as internal controls for the cytoplasmic and nuclear fractions, respectively. ***p < 0.001 (n = 3).

Furthermore, the molecular mechanisms underlying the RNF43 regulation of ccRCC were examined. RNA sequencing was applied to RNF43 knockdown 786-O cells and control 786-O cells (Figs. 5a and 5b; Suppl. Figs. S4a and S4b; Suppl. Tables S5–S7). Relative to their expression in control 786-O cells, 90 genes were upregulated, and 77 genes were downregulated in 786-O cells with RNF43 knockdown (*p* < 0.01, Suppl. Fig. S4a; Suppl. Table S5). Additionally, differentially regulated pathways, including the Hippo pathway, were observed in RNF43-knockdown 786-O cells (Suppl. Table S7). Furthermore, upregulated expression of YAP and YAP target genes (AMOTL1, CTGF, AXL, CYR61, and ANKRD1) was observed (Figs. 5a and 5b; Suppl. Tables S5 and S7). Real-time PCR was performed for validation and confirmed that the expression of YAP was upregulated in RNF43-silenced 786-O or 769-P cells and that RNF43 overexpression decreased YAP expression (Figs. 5c and 5d; Suppl. Figs. S4c and S4d). Additionally, the luciferase promoter activity assay indicated that RNF43 knockdown promoted YAP transcriptional activity in ccRCC cells (Fig. 5e; Suppl. Fig. S4e). However, RNF43-overexpressing ccRCC cells presented decreased transcriptional activity of YAP (Fig. 5f; Suppl. Fig. S4f). Furthermore, higher expression of TEAD1 and YAP target genes, including AXL, ANKRD1, AMOTL1, CYR61, and CTGF, was observed in ccRCC cells with RNF43 knockdown than in RNF43-overexpressing ccRCC cells (Figs. 5c and 5d; Suppl. Figs. S4c and S4d). These findings demonstrated that RNF43 downregulates the expression of YAP and YAP target genes in ccRCC.

YAP is negatively mediated by the Hippo pathway kinase LATS1/2 through phosphorylation, and then p-YAP is sequestered and degraded in the cytoplasm [12]. Only the entry of YAP into the nucleus can upregulate the expression of downstream genes [12]. Nucleoplasm separation-based Western blotting showed that RNF43 knockdown decreased the expression of p-YAP and YAP in the cytoplasm of ccRCC cells but upregulated YAP distribution in the nucleus (Fig. 5g). Conversely, overexpressing RNF43 in ccRCC cells showed opposite effects (Fig. 5h). Additionally, silencing RNF43 increased the expression level of p-LATS1/2 in the cytoplasm of ccRCC cells, but RNF43 overexpression reduced the expression of p-LATS1/2 (Fig. 5g and 5h). These findings indicated that RNF43 inactivates YAP signaling in ccRCC by enhancing YAP phosphorylation through p-LATS1/2 and decreasing the transcription and nuclear localization of YAP.

### RNF43 mediates the malignant characteristics of ccRCC in a YAP-dependent manner

**Figure 6:**
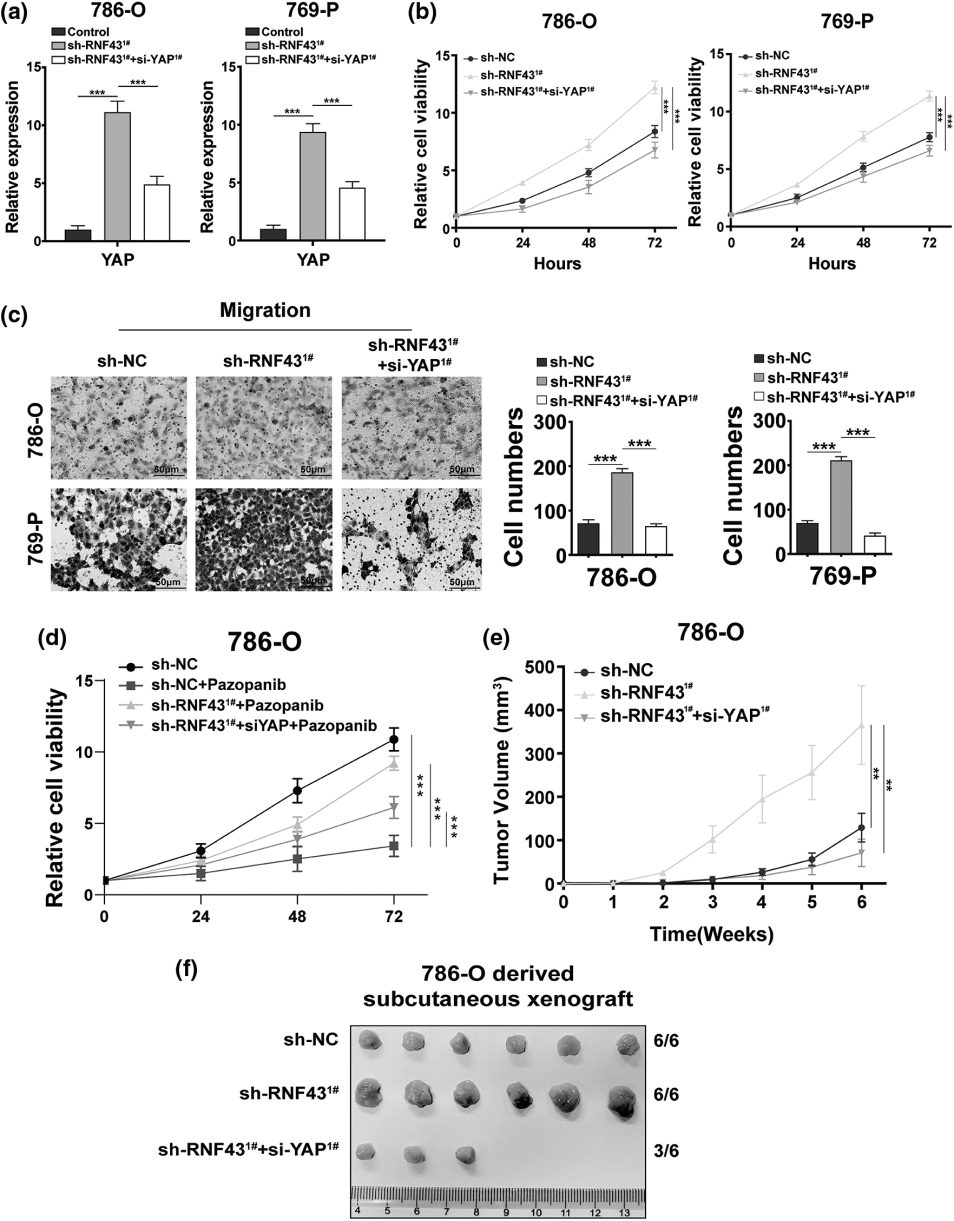
RNF43 mediates the malignant features of ccRCC in a YAP-dependent manner. (a) Real-time PCR was conducted to detect the expression of YAP in RNF43-knockdown 786-O or 769-P cells without or with decreasing YAP by using siRNAs. (b) CCK-8 assays were conducted to detect the proliferation of RNF43-knockdown 786-O or 769-P cells without or with decreasing YAP. (c) Representative images and statistical analysis of the migration assays in RNF43-knockdown 786-O or 769-P cells in the absence or presence of decreasing YAP are shown (scale bar = 200 µm). (d) 786-O cells and RNF43-knockdown 786-O cells without or with decreasing YAP were treated with pazopanib (3 μM) for 36 h, and cell proliferation was evaluated at different times using CCK-8 assays. (e, f) 786-O cells and RNF43-knockdown 786-O cells without or with decreasing YAP were subcutaneously injected into nude mice (n = 6/group). The volumes of tumor xenografts from the different groups were compared at the indicated times (e), and tumor xenografts are shown (f). **p < 0.01 and ***p < 0.001 (n = 3).

Considering the above results, we next investigated whether YAP was required in RNF43-regulated ccRCC. First, YAP was silenced in RNF43-knockdown ccRCC cells (Fig. 6a). Next, CCK-8 proliferation and migration assays were conducted, both demonstrating that decreasing YAP in RNF43-knockdown ccRCC cells abolished the role of silencing RNF43 in promoting the proliferation and migration of ccRCC cells (Figs. 6b and 6c).

Additionally, although RNF43 knockdown facilitated the pazopanib resistance of ccRCC cells, silencing YAP inhibited these effects of RNF43 knockdown (Fig. 6d). Furthermore, an *in vivo* subcutaneous tumor formation assay was employed and showed that RNF43 knockdown enhanced the growth and volume of ccRCC xenografts, while decreasing YAP alleviated the effects of RNF43 knockdown in promoting the tumorigenicity and tumor growth of ccRCC (Figs. 6e and 6f). The findings showed that RNF43 knockdown promotes the malignant features of ccRCC in a YAP-dependent manner.

### RNF43 serves as a potential therapeutic target and helpful prognostic indicator in ccRCC

**Figure 7:**
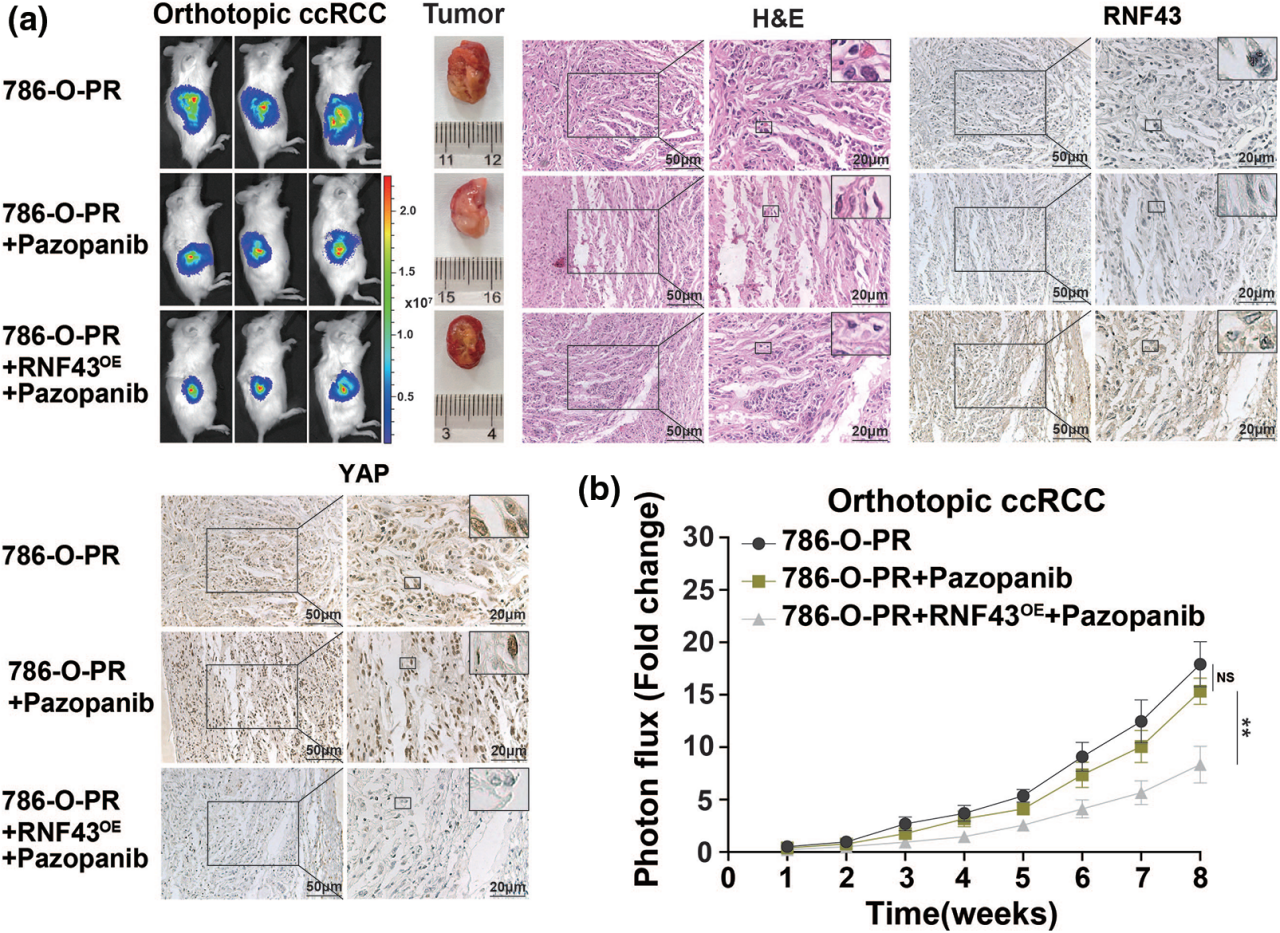
Overexpression of RNF43 inhibits the Pazopanib resistance in orthotopic ccRCC mice (a, b) Stable luciferase-expressing 786-O-PR cells without or with RNF43 overexpression were injected under the renal capsule of mice, and tumor growth was monitored using an *in vivo* imaging system. After 3 weeks, the mice injected with 786-O-PR cells were treated with normal saline or pazopanib (80 mg/kg), and the mice injected with RNF43-overexpressing 786-O-PR cells were treated with pazopanib (80 mg/kg). Images of luciferase intensity, orthotopic xenografts, and H&E and IHC staining of RNF43 and YAP in tumor specimens from different groups are presented (scale bar = 20 µm) (a). Photon flux levels were examined in the different groups of mice, and the results are presented as the fold increase in tumor growth (b). **p < 0.01 (n = 3); NS, no significance.

Given that ccRCC progression occurs even after treatment with targeted drugs and that targeted drug-resistant ccRCC exhibits downregulated RNF43 expression (Figs. 1g and 1h), we next examined whether restoring RNF43 expression could inhibit targeted drug resistance in ccRCC using a pazopanib-resistant orthotopic ccRCC model, which was described in our previous study [17]. In contrast to the naïve and pazopanib-treated 786-O-PR groups, the pazopanib-treated 786-O-PR group with RNF43 overexpression showed slower tumor growth and a smaller tumor volume (Figs. 7a and 7b). Additionally, the pazopanib-treated 786-O-PR group with RNF43 overexpression exhibited lower YAP expression than the other two groups, as determined by IHC assays (Fig. 7a). These findings indicated that restoring RNF43 suppresses pazopanib resistance in ccRCC *in vivo*.

**Figure 8:**
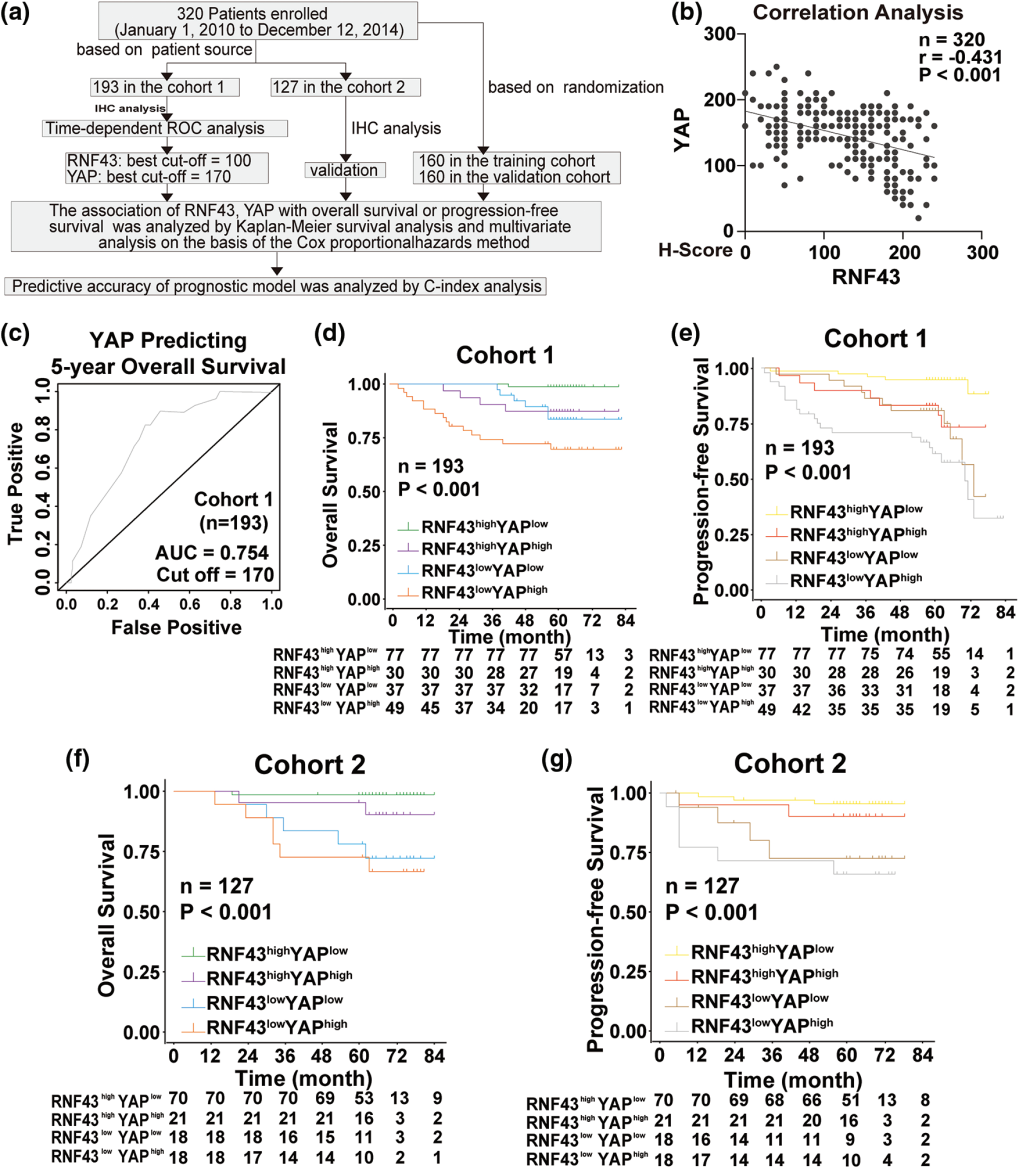
Incorporation of RNF43 and YAP with current clinical prognostic parameters better predicts the prognosis of ccRCC patients. (a) A flow chart of the research design is shown. (b) Correlation analysis between RNF43 and YAP in ccRCC is shown. (c) Time-dependent ROC curve analysis was performed to examine the optimal cut-off value of YAP in cohort 1. (d–g) Kaplan-Meier analyses of OS and PFS were performed in ccRCC patients from cohort 1 (n = 193; d, e) and cohort 2 (n = 127; f, g) **p < 0.01; NS, no significance.

Given the above regulation of YAP by RNF43 in ccRCC, we next examined whether combining the two biomarkers better predicted the ccRCC patient prognosis than either biomarker individually or better than current clinical indicators alone, such as TNM stage and SSIGN (Fig. 8a). First, IHC assays were applied to human ccRCC specimens and showed that RNF43 expression was negatively correlated with YAP expression (Fig. 8b). We next determined the predictive value of the expression of RNF43 and YAP together with clinicopathological characteristics for ccRCC patient prognosis. According to the optimal cut-off values for RNF43 and YAP (Figs. 2b and 8c), ccRCC patients were divided into four groups (RNF43^high^ YAP^low^, RNF43^low^ YAP^high^, RNF43^low^ YAP^low^, and RNF43^high^ YAP^high^). Among the groups, the RNF43^low^ YAP^high^ group presented the highest TNM stages, WHO/ISUP grades, and SSIGN scores and the worst OS and PFS. By contrast, the RNF43^high^ YAP^low^ group exhibited the best levels of clinicopathological characteristics and best OS and PFS (Figs. 8d and 8e; Suppl. Figs. S6a and S6b; Tables 3 and 4). Moreover, these findings were confirmed in the randomized training cohort and validation cohort (Figs. 8f and 8g; Suppl. Figs. S6c and S6d; Suppl. Tables S8 and S9).

**Table 3:** The correlation between expressions of RNF43, YAP and clinicopathologic characteristics of patients with clear cell renal cell carcinoma in cohort 1

Characteristic	RNF43/YAP expression					P* value
	**RNF43^high^ YAP^low^ (n = 77)**	**RNF43^low^ YAP^low^ (n = 37)**	**RNF43^high^ YAP^high^ (n = 30)**	**RNF43^low^ YAP^high^ (n = 49)**	**Sum(193)**	
**Age**						0.165
<60	46	19	13	20	98	
≥60	31	18	17	29	95	
**Gender**						0.022*
Male	59	18	18	34	129	
Female	18	19	12	15	64	
**WHO/ISUP Grading**						0.008*
I–II	60	19	15	30	124	
III–IV	17	18	15	19	69	
**TNM stage**						<0.001*
I–II	74	31	25	28	158	
III–IV	3	6	5	21	35	
**SSIGN**						<0.001*
0–4	75	30	26	35	166	
≥5	2	7	4	14	27	

Note: *: Statistical significance was calculated by chi-square test or fisher’s exact test for categorical/binary measures and ANOVA for continuous measures.

**Table 4:** The correlation between expressions of RNF43, YAP and clinicopathologic characteristics of patients with clear cell renal cell carcinoma in cohort 2

Characteristic	RNF43/YAP expression					P* value
	**RNF43^high^ YAP^low^ (n = 70)**	**RNF43^low^ YAP^low^ (n = 18)**	**RNF43^high^ YAP^high^ (n = 21)**	**RNF43^low^ YAP^high^ (n = 18)**	**Sum (127)**	
**Age**						0.632
<60	34	8	8	6	56	
≥60	36	10	13	12	71	
**Gender**						0.328
Male	53	12	19	13	97	
Female	17	6	2	5	30	
**WHO/ISUP Grading**						0.008*
I–II	52	12	11	6	81	
III–IV	18	6	10	12	46	
**TNM stage**						<0.001*
I–II	70	15	20	13	118	
III–IV	0	3	1	5	9	
**SSIGN**						<0.001*
0–4	70	13	20	15	118	
≥5	0	5	1	3	9	

Note: *: Statistical significance was calculated by chi-square test or fisher’s exact test for categorical/binary measures and ANOVA for continuous measures.

Furthermore, univariate and multivariate Cox regression analyses were performed to show that RNF43 and YAP were independent risk factors for ccRCC patients (Tables 5 and 6; Suppl. Tables S10 and S11). We also investigated whether the incorporation of RNF43 and YAP with either of the current clinical prognostic indicators had greater accuracy than any of these indicators alone in predicting the prognosis of ccRCC patients. Next, Harrell’s c-index analysis was performed, demonstrating that the c-index value of combining RNF43, YAP, and TNM stage or SSIGN was higher than that of any of these indicators alone to predict the OS or PFS of ccRCC patients (Table 7). Therefore, improved prognostic accuracy in assessing the survival of ccRCC patients was achieved by combining the expression of RNF43 and YAP with TNM stage or SSIGN.

**Table 5:** Univariate and multivariate cox regression analysis of RNF43, YAP and clinicopathologic characteristics with overall survival and progression-free survival in cohort 1

Characteristics	Overall survival				Progression-free survival			
	Univariate		Multivariate		Univariate		Multivariate	
	HR (95% CI)	*P* value	HR (95% CI)	*P* value	HR (95% CI)	*P* value	HR (95% CI)	*P* value
**Age (<60 y *vs*. ≥60 y)**	0.892 (0.412–1.928)	0.771			0.870 (0.402–1.882)	0.724		
**Gender (Male *vs*. Female)**	1.513 (0.695–3.295)	0.297			1.536 (0.705–3.35)	0.280		
**WHO/ISUP Grading (1–2 *vs*. 3–4)**	3.651 (1.627–8.194)	**0.002**	1.146 (0.456–2.883)	0.077	3.929 (1.748–8.828)	**0.001**	1.414 (0.572–3.494)	0.054
**TNM stage (1–2 *vs*. 3–4)**	7.868 (3.605–17.173)	**<0.001**	4.295 (2.952–9.532)	**<0.001**	8.143 (3.733–17.763)	**<0.001**	4.508 (2.017–9.182)	**<0.001**
**SSIGN (1–4 *vs*. ≥5)**	11.949 (5.468–26.111)	**<0.001**	9.704 (3.844–24.499)	**<0.001**	11.786 (5.398–25.735)	**<0.001**	8.207 (3.404–19.787)	**<0.001**
**RNF43 expression (Low vs High)**	0.986 (0.98–0.993)	**<0.001**	0.995 (0.987–1.004)	**0.002**	0.987 (0.98–0.993)	**<0.001**	0.995 (0.986–1.003)	**0.003**
**YAP expression (Low *vs*. High)**	1.079 (1.046–1.113)	**<0.001**	1.058 (1.018–1.1)	**0.005**	1.077 (1.045–1.111)	**<0.001**	1.042 (1.005–1.081)	**0.003**

**Table 6:** Univariate and multivariate cox regression analysis of RNF43, YAP expression classifier and clinical characteristicswith overall survival and progression-free survival in cohort 2

Characteristics	Overall survival				Progression-free survival			
	Univariate		Multivariate		Univariate		Multivariate	
	HR (95% CI)	*P* value	HR (95% CI)	*P* value	HR (95% CI)	*P* value	HR (95% CI)	*P* value
**Age (<60 y *vs*. ≥60 y)**	0.581 (0.202–1.676)	0.315			0.584 (0.203–1.683)	0.319		
**Gender (Male *vs*. Female)**	1.24 (0.389–3.953)	0.716			1.281 (0.402–4.084)	0.676		
**WHO/ISUP Grading (1–2 *vs*. 3–4)**	4.973 (1.558–15.872)	**0.007**	6.209 (1.53–25.195)	**0.011**	4.981 (1.56–15.902)	**0.007**	6.159 (1.451–26.142)	**0.014**
**TNM stage (1–2 *vs*. 3–4)**	14.479 (4.982–42.081)	**<0.001**	7.908 (2.394–21.255)	**0.004**	16.108 (5.524–46.968)	**<0.001**	8.655 (0.576–12.241)	**0.002**
**SSIGN (1–4 *vs*. ≥5)**	20.736 (7.168-59.982)	**<0.001**	11.821 (2.537–55.071)	**0.002**	24.440 (8.37–71.363)	**<0.001**	12.145(4.288–69.768)	**0.001**
**RNF43 expression (Low vs High)**	0.984 (0.976–0.993)	**<0.001**	0.991 (0.979–1.003)	**0.002**	0.984 (0.976–0.993)	**<0.001**	0.989 (0.977–1.002)	**0.001**
**YAP expression (Low *vs*. High)**	1.042 (0.999–1.087)	**0.006**	0.996 (0.935–1.062)	**0.009**	1.042 (0.999–1.088)	**0.006**	0.984 (0.922–1.049)	**0.009**

**Table 7:** C-index analysis of the prognostic accuracy of RNF43, YAP and other variables for overall survival and progression-free survival in all cohorts

C-index (95% CI)	Overall survival				Progression-free survival			
	Cohort 1 (n = 193)	Cohort 2 (n = 127)	Training Cohort (n = 160)	Validation Cohort (n = 160)	Cohort 1 (n = 193)	Cohort 2 (n = 127)	Training Cohort (n = 160)	Validation Cohort (n = 160)
**TNM stage**	0.716 (0.697–0.835)	0.701 (0.622–0.781)	0.709 (0.643–0.774)	0.722 (0.632–0.812)	0.69 (0.616–0.775)	0.687 (0.718–0.757)	0.703 (0.620–0.786)	0.704 (0.633–0.814)
**SSIGN**	0.704 (0.624–0.784)	0.729 (0.675–0.783)	0.74 (0.695–0.785)	0.703 (0.624–0.783)	0.681 (0.606–0.756)	0.707 (0.642–0.772)	0.725 (0.6735–0.777)	0.671 (0.624–0.819)
**RNF43**	0.766 (0.674–0.857)	0.797 (0.702–0.892)	0.777 (0.688–0.865)	0.778 (0.670–0.885)	0.748 (0.656–0.840)	0.800 (0.709–0.892)	0.76 (0.671–0.850)	0.756 (0.660–0.852)
**YAP**	0.785 (0.695–0.874)	0.676 (0.553–0.800)	0.76 (0.668–0.852)	0.732 (0.604–0.861)	0.772 (0.670–0.875)	0.671 (0.537–0.805)	0.76 (0.665–0.855)	0.695 (0.551–0.839)
**RNF43 + YAP**	0.796 (0.722–0.870)	0.8 (0.696–0.904)	0.806 (0.705–0.907)	0.783 (0.687–0.878)	0.777 (0.694–0.861)	0.806 (0.735–0.878)	0.792 (0.717–0.867)	0.763 (0.660–0.867)
**RNF43 + YAP + TNM stage**	0.846 (0.785–0.908)	0.876 (0.823–0.929)	0.847 (0.786–0.908)	0.865 (0.797–0.933)	0.839 (0.775–0.903)	0.885 (0.831–0.939)	0.834 (0.769–0.900)	0.865 (0.806–0.924)
**RNF43 + YAP + SSIGN**	0.851 (0.784–0.919)	0.873 (0.803–0.942)	0.833 (0.759–0.906)	0.905 (0.846–0.963)	0.848 (0.775–0.921)	0.895 (0.840–0.950)	0.827 (0.753–0.901)	0.895 (0.836–0.955)

## Discussion

Although many advances have been made in targeted therapy and immune checkpoint inhibitors for advanced ccRCC, tumor progression and a poor prognosis remain clinical barriers. Although many researchers have searched for prognostic indicators and elucidated the mechanisms underlying ccRCC progression [19,20], further studies are needed for validation. In the present study, we identified a novel tumor suppressor and prognostic indicator, RNF43, for ccRCC. Our findings provide a new treatment target and prognostic index for clinical ccRCC patients.

RNF43 is involved in the cell cycle, p53 pathway, and Wnt/β-catenin signaling, linking RNF43 to cell homeostasis and organ development [7]. Thus, the abnormal expression of RNF43 is related to tumorigenesis, such as pancreatic ductal adenocarcinoma, gastric cancer, and colorectal cancer [21]. RNF43 mainly suppresses the Wnt/β-catenin pathway by selectively ubiquitinating Frz receptors or impairing Wnt-target gene expression [7]. Additionally, the Wnt/β-catenin pathway is involved in ccRCC progression [22,23], explaining why RNF43 overexpression inhibits ccRCC. However, a recent study demonstrated that RNF43 facilitates the epithelial-mesenchymal transition (EMT) of lung adenocarcinoma through the ubiquitination and degradation of phosphorylated E-cadherin by activated c-Src [24]. These findings indicate the crucial role of RNF43 in homeostasis regulation. Our present study also identified a novel mechanism by which RNF43 inhibits ccRCC by decreasing YAP transcription and nuclear distribution, independent of the Wnt/β-catenin pathway. However, the antitumorigenic effects of RNF43 and the related mechanisms are far from completely known.

In the clinic, although the monitoring and follow-up of postoperative ccRCC patients largely depend on clinicopathological parameters, including TNM stage [25], these parameters are inadequate for stratifying patients for precision medicine applications. Combining intratumoral biomarkers with clinicopathological features can improve the prognostic accuracy for ccRCC patients [26]. Our study not only demonstrates that RNF43 serves as a prognostic indicator but alsoindicates that combining the expression of RNF43 and YAP with TNM stage or SSIGN achieves improved prognostic ability for ccRCC patients over the ability of any of the indicators alone. To avoid selective bias in the analysis of patients, all the patients with ccRCC were randomly divided into training and validation cohorts. However, further clinical studies are needed to confirm the applicability of the above prognostic indicators.

In conclusion, we demonstrated that RNF43 inhibits ccRCC by inhibiting YAP signaling. Additionally, RNF43 represents a prognostic indicator for ccRCC patients, particularly when combined with YAP and TNM stage or SSIGN score. These findings present a new scientific basis to clarify the mechanisms of ccRCC development and identify prognostic indicators.

## Data Availability

The data that support the findings of this study are available from the corresponding author upon reasonable request.

## Abbreviations:

ccRCC: clear cell renal cell carcinoma
RNF43: ring finger protein 43
YAP: yes-associated protein
TEAD1: TEA domain transcription factor 1
RT-PCR: real-time polymerase chain reaction
GAPDH: glyceraldehyde 3-phosphate dehydrogenase
IHC: immunohistochemistry
DAB: diaminobenzidine
HK-2: human kidney 2
DMEM: Dulbecco’s modified Eagle’s medium
FBS: fetal bovine serum
STR: short tandem repeat
CCK-8: cell counting kit-8
786-O-PR: pazopanib-resistant 786-O cell line
786-O-SR: sunitinib-resistant 786-O cell line
NOD/SCID: nonobese diabetic severe combined immunodeficiency
SD: standard deviation
ROC: receiver operating characteristic
AUC: area under the curve
TNM: Tumor Node Metastasis
WHO/ISUP: World Health Organization/International Society of Urological Pathology
SSIGN: tumor stage, size, grade, and necrosis
OS: overall survival
PFS: progression-free survival
shRNA: short hairpin RNA
LATS1/2: large tumor suppressor 1/2
siRNA: small interfering RNA
H&E: hematoxylin and eosin

## Supplementary Materials

Supplementary Figures and Supplementary Tables are available through a website: https://figshare.com/articles/figure/Supplementary_Tables_and_Figures_pdf/18517796/3.
